# Establishing an Embedded Psychiatry Rotation with Naval Special Warfare: A Win for Both the Education of Military Psychiatry Residents and the Operational Forces

**DOI:** 10.1007/s40596-023-01823-4

**Published:** 2023-07-19

**Authors:** Glennie Leshen, Scott Johnston, Amanda Ries, Lauretta Ziajko, Landon Van Dell, David Nissan

**Affiliations:** 1 Naval Medical Center San Diego, San Diego, CA USA; 2 Naval Special Warfare, San Diego, CA USA

Residency programs strive to train physicians to practice the art of medicine across a wide spectrum of clinical settings, including hospitals, outpatient clinics, and private practices. For the vast majority of physicians practicing in the USA, it is extremely rare to be in communication with their patients’ employers. In most cases, clinicians and their practices have no financial or administrative relationship with patients’ employers. In purely clinical settings, physicians rarely need to think critically about how to communicate with entities other than the patient or family. A different dynamic arises when the physician works for the patient’s employer. In these cases, the clinician must balance the legal and ethical challenges of preserving patient confidentiality. Military clinicians, and especially military psychiatrists, face this challenge daily. A service member’s command may be involved in their lives more than a typical civilian employer. The command must take responsibility for their sailor or marine, and their involvement in clinical care is often essential to keep the US fighting forces medically ready for war. The Accreditation Council for Graduate Medical Education requires programs to address the specific health needs of their specific communities [1]. Thus, learning how to provide care while communicating with the patient’s chain of command is a crucial skill that must be taught in all military medicine training programs, and developing curricula for trainees in these settings should be explored further.

The historical context of military psychiatry provides a foundation and framework for military psychiatry residencies. Since World War I, there has been a push for psychiatrists to be available as close to the front lines as possible [2]. The British government sent psychiatrists out with the troops to help the regimental medical officers with “exhaustion” [3] and found that embedding psychiatrists and psychologists in front-line units led to a dramatic reduction in the number of psychological casualties, which is the term used to describe active-duty service members removed from combat due to mental health issues, such as those experiencing suicidal ideations. The US military experienced a similar result during World War II [3]. By learning from this history, many mental health clinicians in the US Navy currently report directly to an operational unit, which is a small part of a larger military command, rather than a hospital. The experiences during military residency help guide the ethos and culture that military physicians must “serve those who serve” [4]. Military psychiatry residencies place an emphasis on both organization and military leadership, while still training physicians in psychopharmacology and other therapeutic modalities.

These unique aspects of being a military physician are essential learning objectives for all trainees in a military residency. Understanding aspects of service in the US Navy and Marine Corps is important in developing the biopsychosocial formulation, underscoring diagnostic and prognostic considerations. Students from the US military medical school (i.e., Uniformed Services University of the Health Sciences), who were exposed to military culture during their early training years, felt more prepared for operational experiences and deployments than physicians who entered service after their training [5]. Once they are independent, active-duty Navy psychiatrists, psychiatry residents will be working closely with operational commands, where the military’s primary mission of warfighting is at the forefront. This mission includes seeing service members who are still able to function in their full capacity within the military, despite having psychiatric symptomatology. This intersection of symptoms and the workplace forces military psychiatrists to appreciate the occupational health aspects of the field, specifically balancing safety and the needs of the Navy for mission success.

Active-duty psychiatry residents at Naval Medical Center San Diego (NMCSD) have typically worked within the hospital’s adult outpatient clinic, which receives the majority of active-duty military outpatient psychiatry referrals for those patients in the San Diego area who tend to have a high degree of psychiatric comorbidities and treatment resistance. This arrangement leads to psychiatry residents seeing a patient panel of individuals who have been deemed unable to function at their full capacity in the military even before seeing the psychiatry resident for the first time, which gives the military psychiatry residents fewer opportunities to communicate with operational units about whether their service members are capable of meeting the requirements of various missions. This situation is particularly troubling because, once they graduate residency training, they are likely to serve in an operational unit.

The operational command chosen for this rotation, Naval Special Warfare (NSW), is an operationally active combat command consisting of Navy SEALs (i.e., sea, air, and land teams), Special Warfare Combatant Crewmen, and combat support staff (information technology, supply, etc.). The SEALs are an elite special operations combat force. This community has a storied history and a unique culture that distinguishes it from the rest of the Navy and US military [6]. Both medical and mental health personnel work collaboratively within NSW to support patients and ensure physical and psychological optimization of the sailors, thereby supporting the overall mission. These embedded mental health care professionals work very closely with their operational leaders and, in most cases, report directly to them. We saw working with the NSW community as a unique opportunity for our residents to learn more about the embedded care model (as embedded psychiatrists) while also gaining experience in operational medicine.

Therefore, our psychiatry residency program instituted an interdisciplinary, practice-based operational experience to target cognitive and affective outcomes. This experience included teaching residents to formulate appropriate psychiatric treatment plans that synthesize the goals of patients and the needs of their occupational missions. The aim was to do this while working in a multidisciplinary team, with a goal of supporting the cultural competency residency objective of learning to do psychiatry within the military.

## Curriculum Development

Guided by Kern’s six-step process of curriculum development [7], the psychiatry residency program built and designed a rotation that fulfilled the goal of creating a more robust operational experience for our residents (Table 1). Understanding service members’ operational context broadly affects military medicine, as missions hinge on the service members’ psychological and physical functional capabilities. As military psychiatrists may deploy with military units in austere environments, collaborating closely with commanding officers and understanding the needs of operationally active patients are necessary learning outcomes.

**Table 1:** The application of Kern’s six-step model [7] to forming the rotation partnership between the command and psychiatry program

Kern’s six-step model	Application
Problem identification and needs assessment	Residents receive minimal training in working with active-duty service members considered fit for full duty
Targeted needs assessment	Learners = US Navy psychiatry residents
	Learning environment = Limited exposure to patient care within an operational setting
Overarching goal	Increase resident patient hours working with those experiencing mental health issues, but still able to deploy
Educational strategies	Supervised clinical experience
	Group discussion with multidisciplinary case conference, including therapists and medical team
	Reflection through supervision
Barriers to implementation	Electronic Health Record — Differing computer access capabilities
	Administrative — Differing front desk scheduling capabilities
Evaluation and feedback	Residents had increased exposure to patients able to function in their occupation
	Increased practice with advocating for patients with their command leadership

Utilizing the first and second steps of Kern’s model, we completed a needs assessment and received feedback from faculty and program graduates, identifying a gap in the outpatient treatment panel and training of NMCSD psychiatry residents. The prior residency curriculum had operational didactics built in, with monthly field trips to various operational platforms, but did not involve any clinical work or supervision with the embedded providers working for those units. These monthly field trips did not include extended meetings with the non-medical portion of the command. Furthermore, the residency outpatient panel had few patients who remained fit for full duty, which made it difficult to gain cultural competence and familiarity with the challenges of treating active-duty military service members while they continue working in typical military environments. Ideally, the overarching goal would involve the resident outpatient panels having a certain number of patients who were considered operationally capable, with no more than minor duty limitations, but who would still benefit from some type of ongoing psychiatric treatment. Due to the competing needs of a large military hospital, we were unlikely to expand outpatient panels to include this population. Instead, we searched for operational commands in need of psychiatric support. NSW, as operationally active commands near NMCSD with a handful of therapists and no psychiatrist, became an appropriate partnership.

Considering the fifth step of Kern’s model, we identified a few barriers to implementation, including differing computer access and front desk scheduling capabilities. We have addressed a few of these barriers and have streamlined the process.

We began planning for the NSW rotation by reviewing our postgraduate year 3 adult outpatient psychiatry rotation goals and objectives and working to modify this document, creating new objectives that would ensure learners achieve the goal of becoming culturally competent in caring for this unique community. By the end of the NSW rotation, we intend trainees to be prepared for their first positions as Navy psychiatrists across the globe and in various types of commands, including ships, submarines, aviation, and Marine commands. During the rotation, residents not only learn about this special community but also gain experience and knowledge about the importance of culturally competent psychiatric care and the ability to build that background knowledge in other communities that they go on to serve in the military and elsewhere. The goals and objectives that were added to our outpatient rotation document are included in Table 2, developed using Bloom’s taxonomy [8].

**Table 2:** Aspects of Bloom’s taxonomy [8] utilized to develop goals and objectives specific to the Embedded Psychiatry Rotation with Naval Special Warfare (NSW)

Clinical competency	Learning objective	Bloom’s taxonomy	
Patient care	Recognize how the unique culture of the NSW community impacts the delivery of psychiatric care	Understand	Explain ideas
	Identify the differences in psychiatric presentations in this community in comparison to the other outpatient clinics		
	Name the pertinent military instructions relating to psychiatric clearance for duty pertinent to this community		
System-based practice	Explain the organizational structure of NSW, and the placement of medical/psychological care within		
Medical knowledge	Describe the incidence of TBI in the training pipeline and deployed settings, and its overlap with psychiatric symptoms	Analyze	Draw connections among ideas
	Describe the challenges in distinguishing ADHD presentations in adulthood from other possible psychiatric disorders common in this community		
Practice-based learning and improvement	Apply the NSW clinic’s triaging policy to identify higher risk patients	Apply	Use information in new situations
Professionalism	Demonstrate sensitivity to treating NSW patients facing multiple intersecting layers of stigma		
Interpersonal and communication skills	Prepare a case presentation on a challenging clinical and/or administrative patient presentation	Create	Produce new work

Learning objectives sorted in the center by clinical competency, with specific aspects of Bloom’s taxonomy listed to the right. *TBI*, traumatic brain injury; *ADHD*, attention-deficit/hyperactivity disorder

## Resident Feedback

The sixth step of Kern’s model is evaluation and feedback, and we asked our residents about their experience. The residents who have completed the rotation found the experience to be instrumental in improving their ability to understand the operational restrictions of psychotropic medications and balance the needs of the patient and the mission. This rotation provided them with experience in managing waiver requirements (e.g., allowing active-duty patients taking psychotropics to carry firearms and participate in deployments) and conditions commonly found in military outpatient psychiatric practices. For example, residents commonly encountered patients suffering from the sequela of traumatic brain injuries (TBI), a condition commonly seen after recurrent blast exposures, high-intensity training exercises, or frequent combat deployments.

As an example of how this rotation challenged residents to balance patient confidentiality with giving commands need-to-know information, one of the residents who piloted this rotation experienced some difficulties with managing the needs of the patient with the requirements of the command’s mission. The resident had to provide succinct reasoning for why the patient was not appropriate for specific tasks without providing too much patient information. Many of the patient’s stressors stemmed from their interactions with their command, and the resident risked losing patient rapport by providing too much information. While hesitant and pushing for more information, the command acquiesced and chose to follow the psychiatry resident’s recommendations, which allowed the patient to undergo more intensive treatment without interruption from deployments or temporary assignments elsewhere.

Throughout the rotation, the residents would update the command on pertinent mission-affecting developments and counsel patients on how the various treatment modalities may affect their ability to complete their missions. The residents gained practice leading the dialogue with commands on the potential mission effects of various levels and treatment modalities associated with psychiatric care.

## Conclusion

Remaining flexible and looking for opportunities for improvement in a residency program allow for growth and improved learning outcomes. The rotation at NSW engages third-year residents with operational commands while providing them with increased diagnostic and treatment experience with conditions commonly encountered in routine military psychiatric practice, such as post-TBI sequela and attention-deficit/hyperactivity disorder. This rotation is also a win for NMCSD, as it improves warriors’ access to excellent psychiatric care. These experiences prepare the residents for eventual front-line operational duty assignments working in embedded mental health after graduation. Using innovative methods to broaden residents’ training exposure makes psychiatry residencies more successful. We present our training improvement project as a potential model for other programs to establish similar experiences in military and civilian settings, such as police and fire departments.
